# Objective assessment of rosacea erythema severity: a multimodal artificial intelligence framework integrating VISIA® imaging and image-derived tabular features

**DOI:** 10.3389/fmed.2026.1829629

**Published:** 2026-05-11

**Authors:** Jun Zhang, Peijian Hong, Ning Wang, Yang Liu, Yue Wang, LiuQing Chen

**Affiliations:** 1 Department of Dermatology, Wuhan No. 1 Hospital, Wuhan, China; 2 Tongji Hospital, Tongji Medical College, Huazhong University of Science and Technology, Wuhan, China

**Keywords:** AI-assisted diagnosis, clinician’s erythema assessment, feature fusion, multimodal deep learning, self-supervised learning, VISIA facial analysis system

## Abstract

Rosacea is a chronic inflammatory skin disorder characterized by persistent facial erythema. Its clinical assessment relies on the Clinician’s Erythema Assessment (CEA), a subjective scale prone to inter-observer variability. To address the need for diagnostic consistency, this study developed a multimodal artificial intelligence framework for objective CEA grading using standardized VISIA® imaging. We analyzed a retrospective cohort of 1,001 patients. To establish a robust reference standard, three expert dermatologists conducted a multi-step collective audit to reach a unanimous consensus for each case. The framework integrated handcrafted image-derived tabular features with deep learning representations. During training, spatial data augmentations and Focal Loss were implemented to address dataset imbalance and mitigate overfitting. Our results demonstrated that the multimodal fusion model achieved statistically significant improvements over the strong image-only baseline (McNemar’s p=0.031 ; DeLong’s p=0.024 ), yielding a Macro-AUC of 0.902 (95% CI: 0.862–0.937). Furthermore, to address the ordinal nature of the disease severity, the fusion model achieved a Quadratic Weighted Kappa (QWK) of 0.800 and an Intraclass Correlation Coefficient (ICC) of 0.801 (95% CI: 0.720–0.860), indicating excellent alignment with expert consensus. Error analysis revealed that over 95% of misclassifications in intermediate grades (CEA3) were restricted to adjacent categories, reflecting strong clinical safety. Interpretability analysis via layer-wise relevance propagation confirmed the model’s focus on clinically recognized erythema-prone regions.

This study establishes a robust proof-of-concept tool that transforms rosacea assessment from subjective inspection into an objective digital measurement, offering significant translational potential for clinical trials and teledermatology.

## 1 Introduction

Rosacea is a chronic inflammatory skin disorder that primarily affects the central face, manifesting as persistent erythema, flushing, papules, pustules, and telangiectasia. It poses a significant psychosocial and quality-of-life burden worldwide, with prevalence varying across populations and skin types (1, 2). Despite its high prevalence, clinical grading of erythema severity remains largely subjective, relying on visual inspection through clinician-based scales such as the Clinician’s Erythema Assessment (CEA). This subjectivity introduces inter- and intra-observer variability, leading to inconsistent classifications and potentially suboptimal treatment decisions (3, 4).

Over the past decade, extensive research has focused on elucidating the mechanisms underlying rosacea. Advances in immunology, transcriptomics, and genetics have highlighted dysregulated innate immunity, aberrant neurovascular responses, microbial triggers, oxidative stress, and hormonal modulation as key contributors to pathogenesis (5–13). For example, whole-genome sequencing and omics studies have uncovered susceptibility loci and molecular pathways linking neurogenic inflammation with disease severity (6, 9), while single-cell transcriptomics has revealed aberrant fibroblast populations involved in chronic inflammation (5). Other studies have emphasized the role of toll-like receptor signaling (8), estrogen-mediated immune responses (10), and oxidative stress via the NOX2/ROS/NF-κB axis (11). Collectively, these findings have expanded our understanding of rosacea biology, providing novel therapeutic targets and advancing precision dermatology.

Parallel to mechanistic advances, clinical research has described phenotypic heterogeneity and vascular remodeling patterns in rosacea patients. Investigations into eyelid vasculature and aging-related vascular changes have underscored the importance of detailed phenotyping (14, 15). Nevertheless, these descriptive studies rarely extend to quantitative, reproducible grading systems applicable in real-world clinical workflows. While standardized imaging platforms such as the VISIA® facial analysis system provide high-resolution multimodal photographs—including erythema-enhanced views designed to highlight vascular involvement—most clinical applications remain confined to semi-quantitative indices, falling short of objective and automated severity grading (3, 4).

Artificial intelligence (AI), particularly deep learning and self-supervised methods, offers an opportunity to overcome these limitations. Multimodal AI has recently shown tremendous potential across diverse medical domains, ranging from collaborative surgical instrument segmentation (16) and Broad Vision Transformer networks to precision dermatology (17). Recent advances in multimodal learning allow integration of diverse data streams—including imaging and quantitative descriptors—into unified predictive frameworks, which have shown promise in other dermatological contexts (7, 12). However, in rosacea, prior work has predominantly emphasized etiological and mechanistic studies (5–13), with little progress toward leveraging multimodal imaging for clinically actionable severity assessment.

Therefore, the present study aimed to address this gap by developing and validating a multi-stage AI framework for automated CEA grading of rosacea using VISIA multimodal imaging. Our workflow incorporated unsupervised clustering, supervised machine learning with handcrafted and self-supervised features, and deep learning fusion strategies that integrated normal and erythema-enhanced images with quantitative descriptors. We further employed Layer-wise Relevance Propagation (LRP) to evaluate whether model attributions aligned with clinically meaningful erythema distributions, thereby enhancing interpretability and clinical trust (18). This study represents a novel step toward objective, reproducible, and AI-assisted severity grading of rosacea erythema.

## 2 Methods

### 2.1 Study population and design

**Figure 1:**
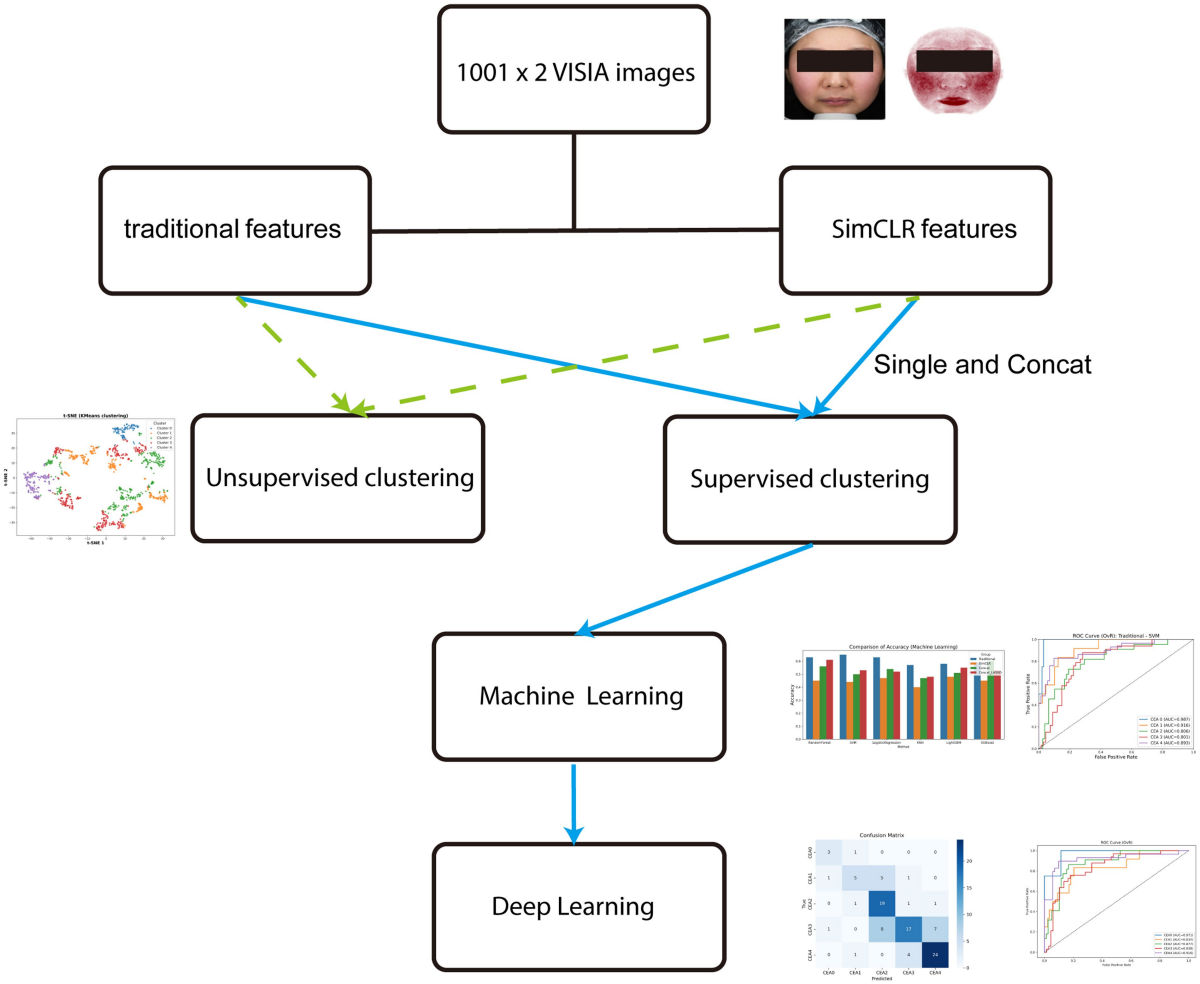
Overall study workflow. The pipeline included patient enrollment, VISIA imaging, expert grading, dataset stratification, feature extraction (traditional vs. SimCLR), unsupervised clustering, supervised machine learning, multimodal deep learning, and explainable AI visualization.

This retrospective study included 1,001 patients who underwent standardized imaging with the VISIA® facial analysis system (Canfield Scientific, USA) at the Department of Dermatology, Wuhan First Hospital, from January 2024 to December 2024, for clinical assessment of rosacea severity. To establish a highly reliable clinical ground truth and minimize individual subjective bias, three associate chief dermatologists conducted a rigorous multi-step collective audit for each case. Any initial disagreements were resolved through joint re-evaluation of the erythema-enhanced views until a unanimous consensus was reached. VISIA normal (M) and red-enhanced (MR) images were paired by patient ID and randomly split into training (*n* = 600), validation (*n* = 300), and independent test (*n* = 101) sets in an approximate 6:3:1 ratio. The detailed distribution of Clinician’s Erythema Assessment (CEA, 0–4 scale) grades across these cohorts is summarized in Table 1. The overall methodological pipeline, including patient enrollment, imaging, expert annotation, and multimodal AI modeling, is illustrated in Figure 1. Given the inherent imbalance in CEA grade distribution, no over- or under-sampling was applied; instead, inverse-frequency class weights were computed based on the training set:


$$w_{c}=\frac{\frac{1}{f_{c}}}{\frac{1}{K}\sum_{k=1}^{K}\frac{1}{f_{k}}}$$

where *fc* is the frequency of class c and *K* is the number of classes (19, 20). These weights were subsequently integrated into the loss functions during model training.

**Table 1:** Dataset distribution.

CEA severity grade	Training (*n* = 600)	Validation (*n* = 300)	Test (*n* = 101)	Total (*N* = 1,001)
Grade 0	22	11	5	38
Grade 1	74	37	12	123
Grade 2	134	68	22	224
Grade 3	197	98	33	328
Grade 4	173	86	29	288

The study protocol was approved by the Institutional Review Board of Wuhan First Hospital (IRB No. [2023] 53). Written informed consent for participation and publication of anonymized images was obtained from all participants. All data were de-identified before analysis. Due to strict institutional ethical guidelines regarding high-resolution facial imagery, granular demographic metadata were restricted to aggregate statistics of clinical severity distributions to prevent the risk of patient re-identification. The study was conducted in accordance with the Declaration of Helsinki and relevant national regulations.

### 2.2 Image preprocessing and dataset preparation

All VISIA normal (M) and erythema-enhanced (MR) images were preprocessed in a standardized manner: images were loaded in RGB format, resized to 240 × 240 pixels using bilinear interpolation, and normalized channel-wise to zero mean and unit variance based on ImageNet statistics (21). For deep learning, M images were retained as three-channel RGB, and MR images were processed as pseudo-color three-channel grayscale inputs. In multimodal settings, M and MR were concatenated along the channel dimension to form six-channel inputs, or fed into separate CNN backbones in dual-branch designs (22).

To enhance model generalization and mitigate overfitting, spatial data augmentations, including random horizontal flipping (*p* = 0.5) and minor affine transformations (up to 10° rotation and 5% translation), were applied exclusively to the training set. Importantly, photometric or color augmentations (e.g., color jitter) were strictly excluded to preserve the physiological ground truth of the erythema intensity.

### 2.3 Model architectures and training

Three supervised learning strategies were implemented in this study. First, traditional machine learning classifiers—including Random Forest, Support Vector Machine (SVM), Logistic Regression, k-Nearest Neighbors (KNN), LightGBM, and XGBoost—were trained using handcrafted features derived from VISIA normal (M) and erythema-enhanced (MR) images (23–27). These handcrafted features included (i) global color statistics (mean and standard deviation of RGB and HSV channels), (ii) texture descriptors such as gray-level co-occurrence matrix (GLCM) features (contrast, correlation, energy, homogeneity), and local binary patterns (LBP), and (iii) quantitative erythema-related indices (red channel ratios, erythema area percentage) (28, 29).

Second, in the self-supervised learning approach, feature embedding were obtained using SimCLR (30). Specifically, M and MR images were passed through a ResNet-50 backbone pre-trained with contrastive learning, and the resulting 2048-dimensional embedding were reduced to 256 dimensions via PCA for stability. These representations were subsequently used as inputs to the same set of classifiers (31).

Third, multimodal deep learning models were constructed with EfficientNet-B1 backbones to evaluate four configurations: (i) MR-only models, (ii) channel-fusion models combining M RGB with MR grayscale images as six-channel inputs, (iii) image plus tabular descriptors (CNN + MLP late fusion), and (iv) dual-branch models where M and MR were processed independently before feature fusion. To address the inherent class imbalance and force the network to focus on hard-to-classify transitional samples (e.g., CEA3), we replaced the standard cross-entropy loss with a Focal Loss function (*γ* = 2.0), scaled by the inverse-frequency class weights defined previously. All deep learning models were trained for up to 40 epochs using the Adam optimizer (initial learning rate = 1 × 10^−4^, weight decay = 1 × 10 ^−4^), ReduceLROnPlateau learning rate scheduling (factor = 0.1, patience = 5), and early stopping (patience = 8) (22).

For feature concatenation experiments, both handcrafted and SimCLR features were combined (Concat), and redundancy was mitigated using LASSO regression, which imposes an ℓ1 penalty to enforce sparsity:


$$\hat{\beta }=\arg\min_{\beta }\{\frac{1}{2n}{|y-\mathrm{X\beta}|}_{2}^{2}+\lambda {|\beta |}_{1}\}$$

The optimal regularization parameter (penalty C, the inverse of λ) was determined to be 0.3594 using a rigorous 5-fold cross-validation strategy (LogisticRegressionCV). This procedure selected a subset of the most informative features before classification, enhancing stability and reducing overfitting (32).

### 2.4 Performance metrics

Model performance on the independent test set was evaluated using accuracy, macro- and weighted-averaged precision, recall, and F1-score, as well as per-class and macro-averaged area under the receiver operating characteristic curve (AUC). The metrics were defined as follows:


$$\text{Accuracy}=\frac{\mathrm{TP}+\mathrm{TN}}{\mathrm{TP}+\mathrm{TN}+\mathrm{FP}+\mathrm{FN}}$$

$$\text{Precision}=\frac{\mathrm{TP}}{\mathrm{TP}+\mathrm{FP}}$$

$$\text{Recall}=\frac{\mathrm{TP}}{\mathrm{TP}+\mathrm{FN}}$$

$$F1=\frac{2\times \text{Precision}\times \text{Recall}}{\text{Precision}+\text{Recall}}$$

where *TP, TN, FP,* and *FN* are true positive, true negative, false positive, and false negative counts, respectively. For multi-class classification, macro-averaged F1-score was computed as


$$\text{Macro}-F1=\frac{1}{K}\sum_{c=1}^{K}F1_{c.}$$

and weighted-averaged F1-score as


$$\text{Weighted}-F1=\frac{\sum_{c=1}^{K}n_{c}\times F1_{c}}{\sum_{c=1}^{K}n_{c}}$$

where *nc* is the number of samples in class *c*. Macro-AUC was defined as the arithmetic mean of per-class AUC values. Furthermore, to explicitly account for the ordinal nature of the 5-level CEA scale, we incorporated the Quadratic Weighted Kappa (QWK) and Mean Absolute Error (MAE), which strictly penalize severe cross-category misclassifications. Clinical inter-rater reliability was assessed using the Intraclass Correlation Coefficient (ICC, two-way random effects, absolute agreement).

To ensure statistical rigor, a 1,000-iteration bootstrap resampling procedure was implemented on the test set to calculate 95% Confidence Intervals (CI) for Accuracy, Macro-F1, Macro-AUC, QWK, and ICC.

### 2.5 Statistical analysis

Continuous variables were expressed as mean ± standard deviation, and categorical variables as counts (percentages). Paired differences in accuracy between models were assessed using McNemar’s test, and AUC differences were compared using DeLong’s method; bootstrap resampling was applied to estimate the sampling distribution of macro-AUC differences, yielding an approximate *z*-statistic and two-sided *p*-value. Statistical significance was set at *p* < 0.05 and exact *p*-values were reported for model comparisons. All analyses were conducted in Python 3.10 (scikit-learn 1.3.0, PyTorch 2.1.0 pingouin 0.5.3). Early stopping and ReduceLROnPlateau learning rate scheduling were applied during training. Random seeds were fixed (Python/NumPy/PyTorch = 42), with deterministic computation enabled (cuDNN benchmark off, deterministic on) (33–36).

## 3 Results

### 3.1 Unsupervised clustering reveals limited separability of single feature sets

Unsupervised clustering was conducted using handcrafted image-based descriptors (“traditional features”) and embedding derived from self-supervised contrastive learning (SimCLR). To quantitatively validate the natural cluster structure of the data, a Silhouette Score analysis was performed varying K from 2 to 6. The data inherently favored fewer clusters (e.g., *K* = 2) rather than forming 5 distinct groups (Supplementary Figure S1).

When forcing K-means clustering (*k* = 5) to align with the 5 clinical CEA grades, both Adjusted Rand Index (ARI) and Normalized Mutual Information (NMI) revealed limited agreement between the clusters and expert labels. Importantly, the Hungarian matching algorithm was strictly utilized to sort the labels for visual alignment in the confusion matrix; as permutation-invariant metrics, the raw and matched ARI/NMI scores remained mathematically identical. Neither approach produced grade-pure clusters. These findings empirically confirm that unsupervised methods alone are insufficient for fine-grained ordinal severity grading, fundamentally justifying the necessity of our supervised multimodal framework (Supplementary Figure S1).

### 3.2 Supervised machine learning shows superior performance of traditional features over self-supervised embedding

**Figure 2:**
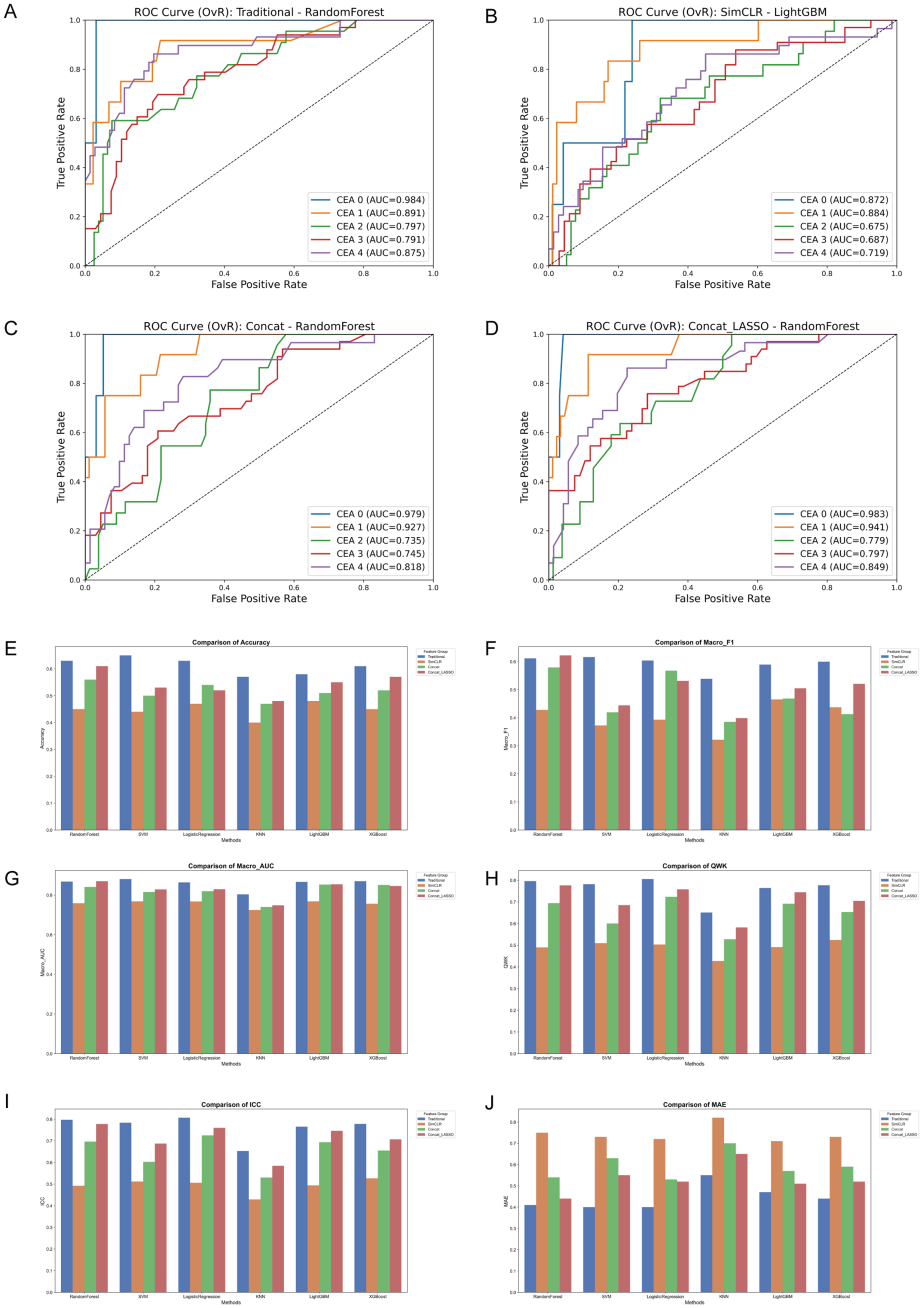
Supervised machine learning performance across feature families. **(A–D)** Multi-class ROC curves (one-vs-rest strategy) for top-performing classifiers using traditional, SimCLR, Concat, and LASSO-regularized Concat feature sets. **(E–J)** Comparative performance analysis across six machine learning algorithms. Metrics include **(E)** accuracy, **(F)** macro-F1, **(G)** macro-AUC, **(H)** quadratic weighted Kappa (QWK), **(I)** intraclass correlation coefficient (ICC), and **(J)** mean absolute error (MAE). Traditional handcrafted features and LASSO-optimized ensembles demonstrated superior capability in capturing ordinal disease severity compared to raw self-supervised embedding.

Supervised classification was performed using multiple algorithms across handcrafted features, SimCLR embedding, and their combinations (Concat and Concat_LASSO). A LASSO-based feature selection step (optimal penalty *C* = 0.3594) was applied to reduce redundancy (Supplementary Table S1 and Figure 2).

Overall, handcrafted quantitative descriptors yielded significantly stronger performance than self-supervised SimCLR embedding in predicting CEA grades. Using traditional features, Support Vector Machine (SVM) and Random Forest emerged as the top-performing classifiers, achieving Accuracies of 0.650[95% CI: 0.560–0.740] and 0.630[95% CI: 0.530–0.720], alongside Macro-AUCs of 0.881[95% CI: 0.836–0.918] and 0.868[95% CI: 0.817–0.911], respectively. Furthermore, evaluating these traditional models on ordinal severity metrics revealed excellent clinical agreement: Logistic Regression and Random Forest achieved Intraclass Correlation Coefficients (ICC) of 0.807 and 0.797, and Quadratic Weighted Kappa (QWK) scores of 0.805 and 0.796, proving the strong clinical relevance of the handcrafted features.

In contrast, SimCLR embedding alone demonstrated weaker separability, with Macro-AUCs consistently below 0.77. While direct feature concatenation (Concat) introduced noise and slightly degraded performance across most linear classifiers, applying LASSO regularization (Concat_LASSO) effectively filtered redundancy and restored stability. For instance, Random Forest trained on Concat_LASSO features achieved a Macro-F1 of 0.622[95% CI: 0.444–0.743] and a Macro-AUC of 0.870. Nevertheless, handcrafted features remained the most robust standalone predictors in traditional supervised settings, establishing a strong foundation for multimodal deep learning integration

In summary, handcrafted features remained the strongest predictors in supervised settings, whereas SimCLR embedding required integration strategies to be effective.

### 3.3 Deep learning analysis: image-only CNN provides a strong baseline while multimodal fusion with LASSO-selected features yields incremental gains

**Figure 3:**
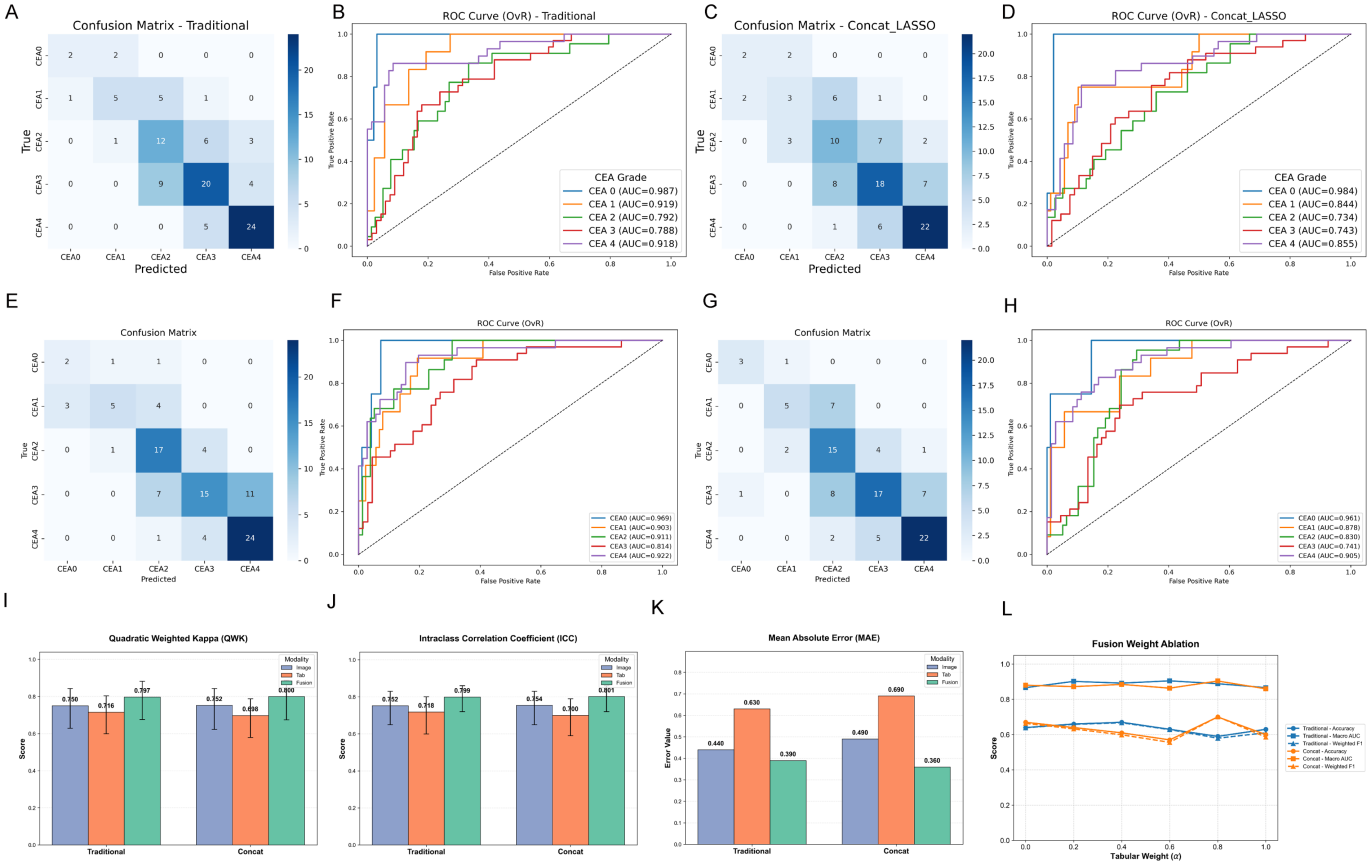
Deep learning results for unimodal and multimodal fusion. **(A–D)** Confusion matrices and ROC curves for the image-only baseline and **(E–H)** the best-performing multimodal fusion models, demonstrating the impact of integrating handcrafted tabular features. **(I–K)** Statistical comparison of image-only, tabular-only, and fusion models across ordinal-sensitive metrics: **(I)** QWK, **(J)** ICC, and **(K)** MAE. Error bars represent 95% confidence intervals (CI) derived from 1,000 bootstrap iterations. The multimodal fusion approach achieved statistically significant superiority over unimodal baselines (*p* < 0.05). **(L)** Ablation study of the tabular fusion weight ( α ) from 0.0 to 1.0 with a 0.2 step interval, highlighting the optimal balance between spatial and quantitative representations. **(J)** Ablation experiments varying the fusion weight *α*, showing stable performance for 0.2–0.8 and optimal results at α = 0.8.

Deep learning models were systematically evaluated to test whether integrating LASSO-selected tabular descriptors could further enhance CNN image-only baselines (Supplementary Table S2 and Figure 3).

**Figure 4:**
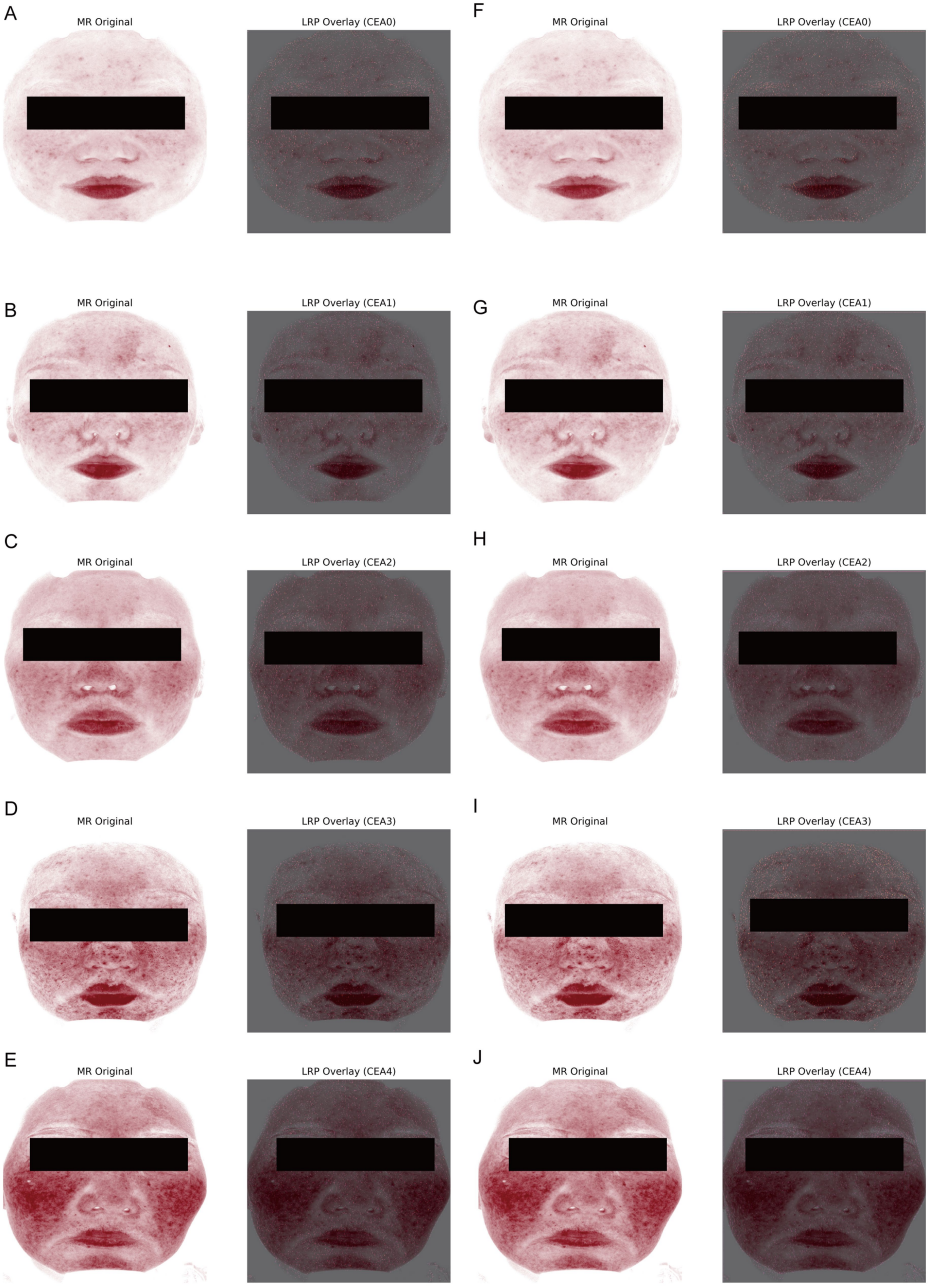
LRP visualizations of model relevance. **(A–E)** Representative LRP heatmaps overlaid on VISIA erythema-enhanced images (CEA0–CEA4) for the traditional fusion model (CNN + tabular features, α = 0.8), illustrating that high-relevance regions correspond to clinically recognized erythema distributions. **(F–J)** Representative LRP heatmaps for the Concat fusion model (*α* = 0.2), showing consistent attribution patterns across central facial regions, with progressively stronger and broader activation in higher CEA grades.

The implementation of spatial data augmentation and Focal Loss (*γ* = 2.0) established an exceptionally strong image-only CNN baseline (Accuracy = 0.630[95% CI: 0.530–0.720], Macro-AUC = 0.826[95% CI: 0.700–0.901]). Crucially, the multimodal late fusion of CNN features with LASSO-selected tabular descriptors (optimal at *α* = 0.8, Figure 4A) achieved statistically significant improvements over the image-only baseline. The best fusion model reached an Accuracy of 0.700[95% CI: 0.610–0.780] and a Macro-AUC of 0.904[95% CI: 0.860–0.938].

Statistical testing confirmed the superiority of the multimodal approach: McNemar’s test demonstrated that the fusion model corrected significantly more errors than it introduced compared to the image-only model (*p* = 0.031), and DeLong’s test confirmed a significant gain in Macro-AUC (*p* = 0.024) (Supplementary Table S3).

Furthermore, to explicitly assess the model’s capability in capturing the ordinal progression of erythema severity, we evaluated the Quadratic Weighted Kappa (QWK) and Intraclass Correlation Coefficient (ICC) (Figure 3). The fusion model achieved a remarkable QWK of 0.800[95% CI: 0.675–0.888] and a clinical ICC of 0.801[95% CI: 0.720–0.860], indicating excellent, near-perfect agreement with the expert consensus panel. The Mean Absolute Error (MAE) was also substantially reduced to 0.360.

A targeted error analysis for the intermediate, hard-to-classify CEA3 grade via the normalized confusion matrix revealed a strong clinical safety profile. Despite a lower exact recall for CEA3, over 95% of its misclassifications were strictly assigned to adjacent categories (i.e., predicted as CEA2 or CEA4), with zero severe cross-level errors (e.g., misclassified as CEA0 or CEA1). This adjacent misclassification pattern mirrors real-world inter-observer variability in transitional cases, proving the model effectively internalized the ordinal severity of the disease.

### 3.4 LRP visualizations highlight relevance in clinically consistent erythema regions

Layer-wise Relevance Propagation (LRP) was applied to the best-performing models to evaluate whether model attributions aligned with clinical erythema patterns (Figure 4). Both fusion models consistently emphasized erythema-prone regions, including the cheeks, nose, and perioral area—hallmarks of rosacea. With increasing CEA grades, LRP maps exhibited expanded and intensified attribution patterns, paralleling clinical severity progression. In CEA0 cases, attributions were minimal and largely confined to background skin texture. These findings confirm that multimodal CNNs not only achieved strong quantitative performance but also directed relevance toward biologically and clinically meaningful areas.

## 4 Discussion

Rosacea severity grading remains a critical unmet need in dermatology, as current assessments rely heavily on subjective interpretation, leading to inconsistent classifications and delays in treatment escalation (1, 4). In this study, we developed a multi-stage artificial intelligence (AI) framework based on VISIA high-resolution imaging, integrating handcrafted quantitative descriptors, self-supervised representations, and multimodal deep learning strategies. Our findings demonstrated that the integration of spatial data augmentation, Focal Loss, and multimodal late fusion of image and tabular descriptors yielded statistically significant improvements (*p* < 0.05) over strong image-only baselines. These results highlight the complementary value of clinically interpretable features and deep learning representations, supporting the role of AI in reducing variability in rosacea grading.

From a clinical perspective, automated erythema assessment offers substantial translational potential. In outpatient practice, AI-derived scores can provide dermatologists with reproducible quantitative indices, enabling earlier intervention for patients at risk of progression (37). In teledermatology, objective computerized grading facilitates reliable remote monitoring, reducing patient burden and expanding access to specialist care (3). In clinical research, standardized automated endpoints could improve the consistency of outcome measures across multicenter trials, thereby accelerating the evaluation of emerging therapies and cosmetic interventions targeting erythema (15).

A notable strength of this study lies in its demonstration of explainability. Layer-wise Relevance Propagation (LRP) visualizations confirmed that model relevance maps corresponded to clinically recognized erythema distributions, a key step in building physician trust and supporting clinical adoption (38). Moreover, the observed improvements with multimodal fusion, although modest, suggest opportunities for extending the framework to incorporate additional VISIA channels (e.g., ultraviolet, brown spots, porphyrins) and multi-scale descriptors beyond erythema (14). Moving from unimodal severity grading to multi-label learning could further enable comprehensive evaluation of vascular remodeling, pigmentation, pore density, and sebum production, contributing to the creation of a digital “skin twin” for rosacea patients (5).

Several limitations should be acknowledged. First, this was a single-center retrospective study, which may limit generalizability. Future prospective, multi-center Phase 2 trials are required to directly compare the AI’s diagnostic performance against human readers of varying experience levels (e.g., junior versus senior dermatologists). Second, due to strict ethical guidelines regarding facial image de-identification, granular demographic metadata were restricted to aggregate statistics, limiting subgroup demographic analyses. Third, the inherent imbalance in CEA grade distribution was largely mitigated using Focal Loss, but severe cases remained relatively underrepresented. Fourth, analyses were restricted to VISIA-derived normal and erythema-enhanced images, without incorporating other modalities such as dermoscopy or reflectance confocal microscopy (RCM). Finally, explainability analyses were limited to qualitative LRP heatmaps without systematic quantitative validation. Because rosacea erythema presents as a diffuse vascular condition without sharp anatomical boundaries, obtaining pixel-level quantitative “erythema masks” from clinicians is highly subjective and clinically unfeasible. Thus, qualitative alignment with clinically recognized erythema distributions remains the most reliable standard for interpretability in this context.

## 5 Conclusion

This study developed a multi-stage AI framework integrating VISIA® high-resolution imaging with image-derived tabular features, self-supervised representations, and multimodal deep learning for automated grading of rosacea erythema severity. While unsupervised clustering demonstrated limited separability, supervised machine learning confirmed the strong discriminatory power of handcrafted features. Importantly, deep learning experiments confirmed that incorporating spatial data augmentation and Focal Loss, alongside multimodal late fusion, delivered statistically significant improvements (*p* < 0.05) over unimodal baselines. The achievement of an ICC exceeding 0.80 and localized adjacent misclassification patterns confirm that the model effectively captures the ordinal clinical severity of the disease. Furthermore, LRP visualizations revealed close alignment between model relevance maps and clinically recognized erythema distributions, improving interpretability.

The significance of this work lies in establishing an objective and reproducible tool for rosacea severity assessment, complementing dermatologist evaluation and reducing inter-observer variability.

The findings suggest that AI-enhanced imaging may serve as a valuable adjunct in routine dermatology practice, teledermatology, and clinical trials, particularly in scenarios where diagnostic consistency is most critical

## Data Availability

The raw data supporting the conclusions of this article will be made available by the authors, without undue reservation.

## Supplementary material

SUPPLEMENTARY FIGURE S1 Unsupervised discovery and cluster validation analysis. **(A–D)** t-SNE visualizations of the natural data structure using **(A,B)** traditional quantitative descriptors and **(C,D)** SimCLR embedding, colored by K-means clusters (*k* = 5) and clinical CEA grades. **(E)** External agreement metrics (Adjusted Rand Index [ARI] and Normalized Mutual Information [NMI]) comparing clustering purity. Hungarian matching was utilized for visualization alignment only and did not alter permutation-invariant metrics. **(F)** Optimal cluster discovery via Silhouette Analysis for *K* = 2 to 6. The results confirm that the data does not naturally partition into five distinct clusters, thereby justifying the necessity of the supervised deep learning framework for fine-grained clinical grading.

## References

[1] Alexis AF Callender VD Baldwin HE Desai SR Rendon MI Taylor SC. Global epidemiology and clinical spectrum of rosacea, highlighting skin of color: review and clinical practice experience. J Am Acad Dermatol. (2018) 80:1722–1729.e7. doi: 10.1016/j.jaad.2018.08.049, 30240779

[2] Barakji YA Rønnstad ATM Christensen MO Zachariae C Wienholtz NKF Halling A-S . Assessment of frequency of Rosacea subtypes in patients with Rosacea: a systematic review and Meta-analysis. JAMA Dermatol. (2022) 158:617–25. doi: 10.1001/jamadermatol.2022.0526, 35385049 PMC8988027

[3] Thiboutot D Anderson R Cook-Bolden F Draelos Z Gallo RL Granstein RD . Standard management options for rosacea: the 2019 update by the National Rosacea Society expert committee. J Am Acad Dermatol. (2020) 82:1501–10. doi: 10.1016/j.jaad.2020.01.077, 32035944

[4] Van Zuuren EJ. Rosacea. N Engl J Med. (2017) 377:1754–64. doi: 10.1056/NEJMcp1506630, 29091565

[5] Chen M Yang L Zhou P Jin S Wu Z Tan Z . Single-cell transcriptomics reveals aberrant skin-resident cell populations and identifies fibroblasts as a determinant in rosacea. Nat Commun. (2024) 15:8737. doi: 10.1038/s41467-024-52946-7, 39384741 PMC11464544

[6] Deng Z Chen M Zhao Z Xiao W Liu T Peng Q . Whole genome sequencing identifies genetic variants associated with neurogenic inflammation in rosacea. Nat Commun. (2023) 14:3958. doi: 10.1038/s41467-023-39761-2, 37402769 PMC10319783

[7] Geng RSQ Bourkas AN Mufti A Sibbald RG. Rosacea: pathogenesis and therapeutic correlates. J Cutan Med Surg. (2024) 28:178–89. doi: 10.1177/12034754241229365, 38450615 PMC11015710

[8] Li G Tang X Zhang S Deng Z Wang B Shi W . Aging-conferred SIRT7 decline inhibits Rosacea-like skin inflammation by modulating toll-like receptor 2–NF-κB signaling. J Invest Dermatol. (2022) 142:2580–2590.e6. doi: 10.1016/j.jid.2022.03.026, 35413292

[9] Mao R Li J. Construction of a molecular diagnostic system for neurogenic rosacea by combining transcriptome sequencing and machine learning. BMC Med Genet. (2024) 17:232. doi: 10.1186/s12920-024-02008-0, 39272052 PMC11396881

[10] Tang J Chen P Huang C Wang W Wang B Shi W . 17β-estradiol promotes LL37-induced rosacea-like skin inflammation via G protein-coupled estrogen receptor 30. J Dermatol Sci. (2025) 119:101–11. doi: 10.1016/j.jdermsci.2025.05.005, 40670220

[11] Wang Y Long L Chen M Li J. Oxidative stress mediated by the NOX2/ROS/NF-κB signaling axis is involved in rosacea. Arch Dermatol Res. (2025) 317:505. doi: 10.1007/s00403-025-03898-5, 40014137

[12] Yang F Wang L Song D Zhang L Wang X Du D . Signaling pathways and targeted therapy for rosacea. Front Immunol. (2024) 15:1367994. doi: 10.3389/fimmu.2024.1367994, 39351216 PMC11439730

[13] Zhang Y Huang Y Wang B Shi W Hu X Wang Y . Integrated omics reveal the molecular characterization and pathogenic mechanism of Rosacea. J Invest Dermatol. (2024) 144:33–42.e2. doi: 10.1016/j.jid.2023.05.028, 37437773

[14] Guihua L Wencai J Shan D Yanzhu J Baek D Lee Y . A study on the difference in aging characteristics of sensitive and non-sensitive skin. Skin Res Technol. (2025) 31:e70172. doi: 10.1111/srt.70172

[15] Peng Y Wang B Mao M Li J Shi W Zhao H . Clinical characteristics of the well-defined upper eyelid vascular network pattern in patients with rosacea. Int J Dermatol. (2024) 63:337–44. doi: 10.1111/ijd.16946, 38197322

[16] Song W Wang X Jiang Y Li S Hao A Hou X . Expressive 3D facial animation generation based on local-to-global latent diffusion. IEEE Trans Vis Comput Graph. (2024) 30:7397–407. doi: 10.1109/TVCG.2024.3456213, 39255115

[17] Shi S Liu W. B2-ViT net: broad vision transformer network with broad attention for seizure prediction. IEEE Trans Neural Syst Rehabil Eng. (2024) 32:178–88. doi: 10.1109/TNSRE.2023.3346955, 38145523

[18] Bach S Binder A Montavon G Klauschen F Müller K-R Samek W. On pixel-wise explanations for non-linear classifier decisions by layer-wise relevance propagation. PLoS One. (2015) 10:e0130140. doi: 10.1371/journal.pone.0130140, 26161953 PMC4498753

[19] Tan J Liu H Leyden JJ Leoni MJ. Reliability of clinician erythema assessment grading scale. J Am Acad Dermatol. (2014) 71:760–3. doi: 10.1016/j.jaad.2014.05.044, 24999270

[20] Polat G Çağlar ÜM Temizel A. Class distance weighted cross entropy loss for classification of disease severity. Expert Syst Appl. (2025) 269:126372. doi: 10.1016/j.eswa.2024.126372

[21] Deng J Dong W Socher R Li L-J Li K Fei-Fei L. ImageNet: a large-scale hierarchical image database. In: 2009 IEEE Conference on Computer Vision and Pattern Recognition. (2009). 248–255.

[22] Tan M Le QV. EfficientNet: Rethinking Model Scaling for Convolutional Neural Networks. (2020) doi: 10.48550/arXiv.1905.11946

[23] Ishwaran H Kogalur UB Blackstone EH Lauer MS. Random survival forests. Ann Appl Stat. (2008) 2, 841–860. doi: 10.1214/08-AOAS169

[24] Cortes C Vapnik V. Support-vector networks. Mach Learn. (1995) 20:273–97. doi: 10.1007/BF00994018

[25] Nelder JA Wedderburn RWM. Generalized linear models. Journal of the Royal Statistical Society Series A (General). (1972) 135:370–84. doi: 10.2307/2344614

[26] Cover T Hart P. Nearest neighbor pattern classification. IEEE Trans Inf Theory. (1967) 13:21–7. doi: 10.1109/TIT.1967.1053964

[27] Chen T Guestrin C. "XGBoost: a scalable tree boosting system". In: *Proceedings of the 22nd ACM SIGKDD International Conference on Knowledge Discovery and Data Mining*. KDD ‘16. New York, NY: Association for Computing Machinery (2016).

[28] V BS Unnikrishnan A Balakrishnan K. Gray level co-occurrence matrices: generalisation and some new features. (2012) [Epub ahead of print]. doi: 10.48550/arXiv.1205.4831

[29] Su Z Pietikäinen M Liu L. From local binary patterns to pixel difference networks for efficient visual representation learning. (2023) [Epub ahead of print]. doi: 10.48550/arXiv.2303.08414 37527290

[30] Chen T Kornblith S Norouzi M Hinton G. A simple framework for contrastive learning of visual representations. (2020) [Epub ahead of print]. doi: 10.48550/arXiv.2002.05709

[31] Alexis A Callender V Baldwin H. Global epidemiology and clinical spectrum of rosacea, highlighting skin of color: Review and clinical practice experience. J. Am. Acad. Dermatol. (2018) 80, 1722–1729.e7. 10.1016/j.jaad.2018.08.049 30240779

[32] Mei Z Shi Z. On LASSO for high dimensional predictive regression. (2024) [Epub ahead of print]. doi: 10.48550/arXiv.2212.07052

[33] Paszke A Gross S Massa F Lerer A Bradbury J Chanan G PyTorch: an imperative style, high-performance deep learning library. (2019) [Epub ahead of print]. doi: 10.48550/arXiv.1912.01703

[34] Pedregosa F Varoquaux G Gramfort A Michel V Thirion B Grisel O Scikit-learn: machine learning in Python. (2018) [Epub ahead of print]. doi: 10.48550/arXiv.1201.0490

[35] Chetlur S Woolley C Vandermersch P Cohen J Tran J Catanzaro B . cuDNN: efficient primitives for deep learning. (2014) [Epub ahead of print]. doi: 10.48550/arXiv.1410.0759

[36] Al-Kababji A Bensaali F Dakua SP. Scheduling techniques for liver segmentation: ReduceLRonPlateau vs OneCycleLR. (2022) [Epub ahead of print]. doi: 10.48550/arXiv.2202.06373

[37] Pan Y Jia K Yan S Jiang X. Effectiveness of VISIA system in evaluating the severity of rosacea. Skin Res Technol. (2022) 28:740–8. doi: 10.1111/srt.13194, 35818722 PMC9907647

[38] Choy SP Kim BJ Paolino A Tan WR Lim SML Seo J . Systematic review of deep learning image analyses for the diagnosis and monitoring of skin disease. NPJ Digit Med. (2023) 6:180. doi: 10.1038/s41746-023-00914-8, 37758829 PMC10533565

