# Supplementary figures and images for: Objective assessment of rosacea erythema severity: a multimodal artificial intelligence framework integrating VISIA® imaging and image-derived tabular features
Source: Front Med (Lausanne). 2026 May 11;13:1829629. doi: 10.3389/fmed.2026.1829629 (PMC13227112; doi:10.3389/fmed.2026.1829629)

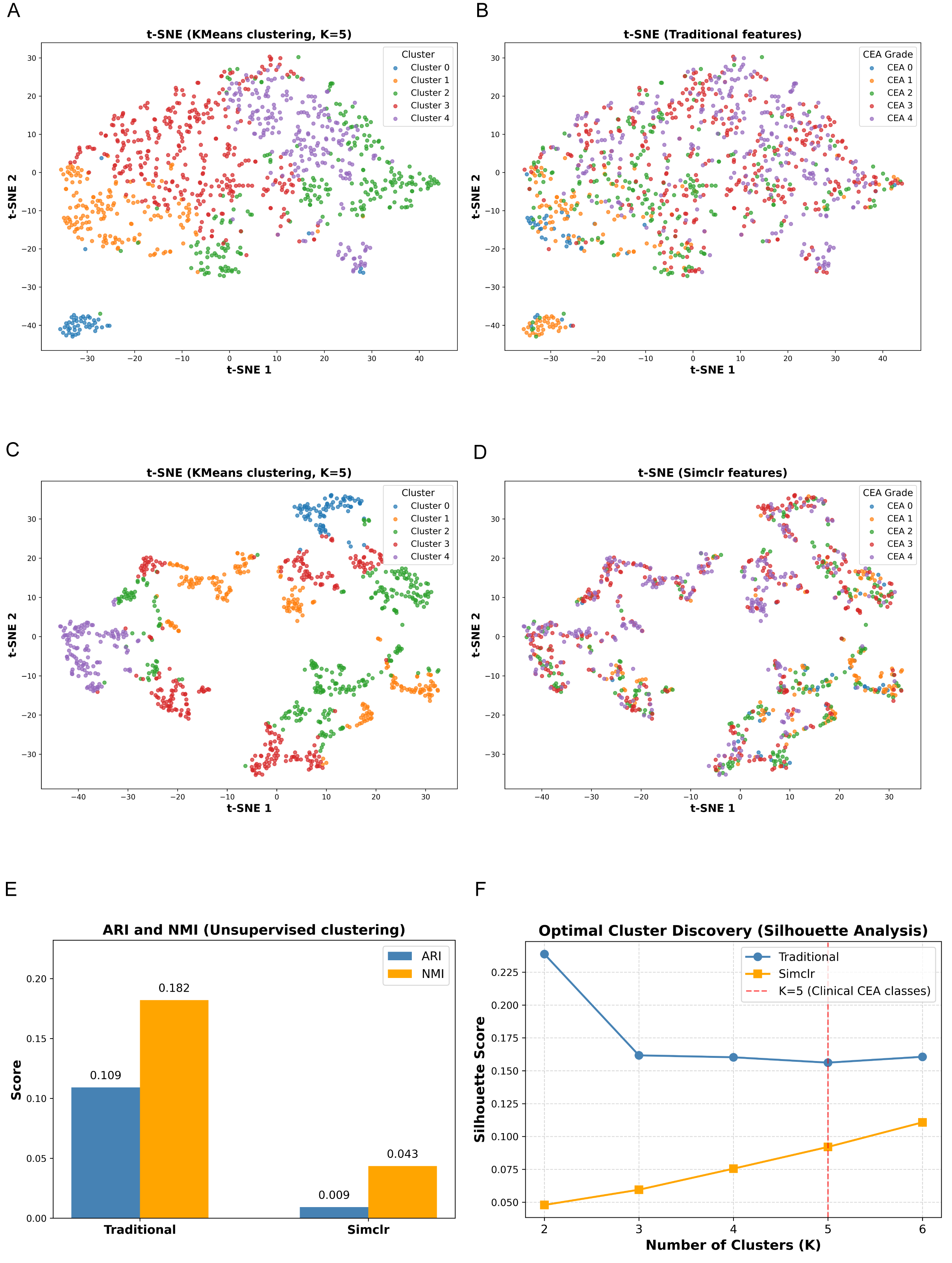

Supplement: SUPPLEMENTARY FIGURE S1 — Unsupervised discovery and cluster validation analysis. (A–D) t-SNE visualizations of the natural data structure using (A,B) traditional quantitative descriptors and (C,D) SimCLR embedding, colored by K-means clusters (k = 5) and clinical CEA grades. (E) External agreement metrics (Adjusted Rand Index [ARI] and Normalized Mutual Information [NMI]) comparing clustering purity. Hungarian matching was utilized for visualization alignment only and did not alter permutation-invariant metrics. (F) Optimal cluster discovery via Silhouette Analysis for K = 2 to 6. The results confirm that the data does not naturally partition into five distinct clusters, thereby justifying the necessity of the supervised deep learning framework for fine-grained clinical grading. [file Image_1.TIF]
